# Supplementary material for: Engineering and Characterization of a Long-Half-Life Relaxin Receptor RXFP1 Agonist
Source: Mol Pharm. 2024 Aug 12;21(9):4441–9. doi: 10.1021/acs.molpharmaceut.4c00368 (PMC11372834; doi:10.1021/acs.molpharmaceut.4c00368)
Supplement: Supplementary file 1 — mp4c00368_si_001.pdf [file mp4c00368_si_001.pdf]

Supporting Information

Engineering and characterization of a long half-life relaxin receptor RXFP1 agonist

Authors: Sarah C. Erlandson[1], Jialu Wang[2], Haoran Jiang[2], James Osei-Owusu[1], Howard A. Rockman[2,3], Andrew C. Kruse[1*]

Affiliations:

[1]Department of Biological Chemistry and Molecular Pharmacology, Blavatnik Institute, Harvard Medical School, Boston, Massachusetts 02115, USA.

[2]Department of Medicine, Duke University Medical Center, Durham, North Carolina 27710, USA.

[3]Department of Cell Biology, Duke University Medical Center, Durham, North Carolina 27710, USA.

*Correspondence and requests for materials should be addressed to andrew_kruse@hms.harvard.edu

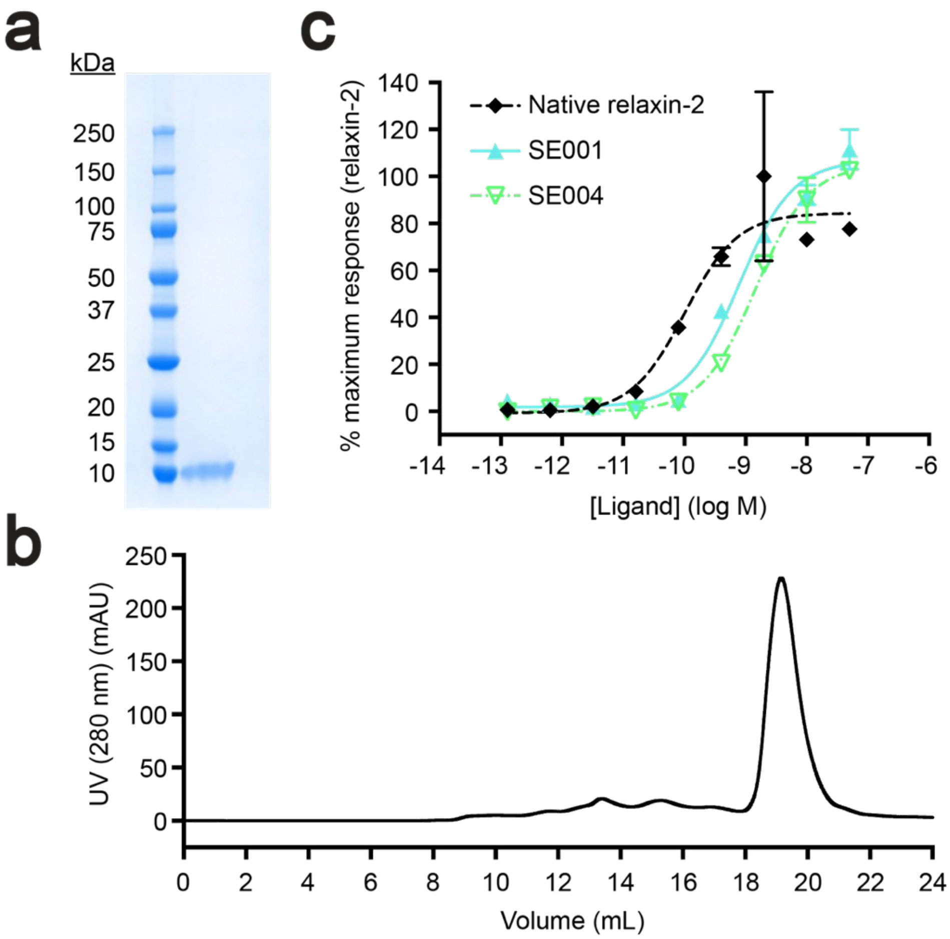

Figure S1: Purification and characterization of single-chain relaxin-2. a, Coomassie-stained SDS-PAGE gel for His-tagged single-chain relaxin-2 (SE001). **b**, Size exclusion chromatography profile for His-tagged single-chain relaxin-2 (SE001). **c**, CRE-SEAP G_s signaling assay with human RXFP1 for His-tagged single-chain relaxin-2 (SE001) and His-tagged, protein C-tagged single-chain relaxin-2 (SE004). Data are normalized to the native relaxin-2 response and are mean ± s.e.m. from technical triplicates.

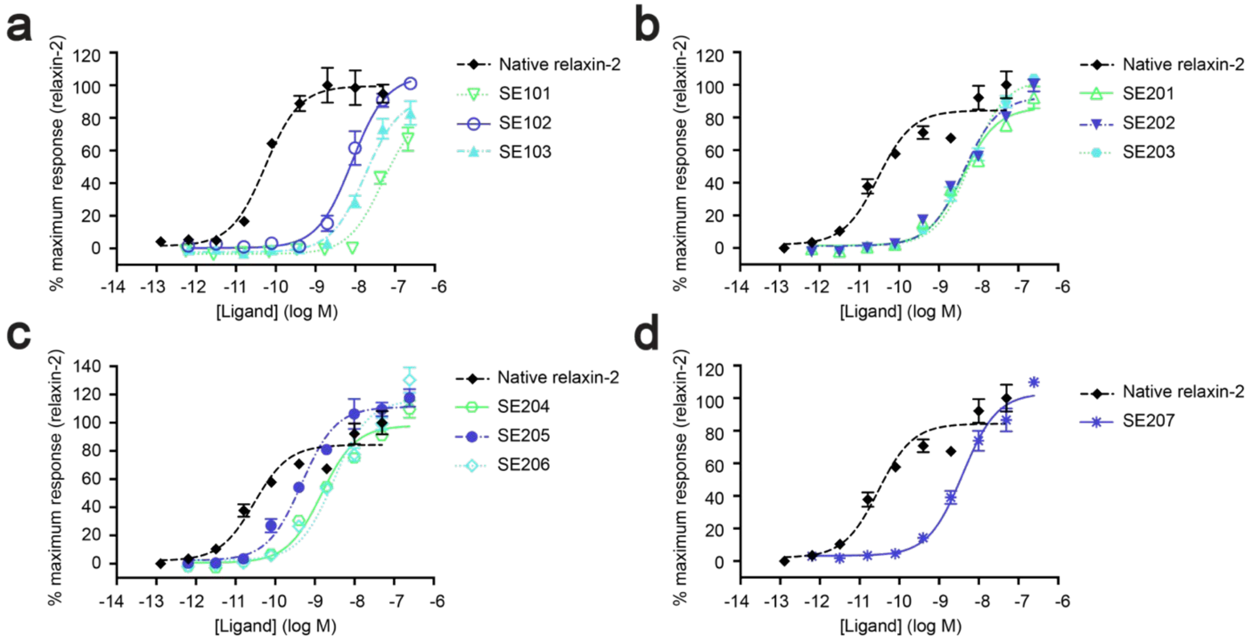

Figure S2: Optimizations of Fc–relaxin-2 fusions. a-d, CRE-SEAP G_s signaling data for human RXFP1 using native relaxin-2 compared to SE101–SE103 (**a**), SE201–SE203 (**b**), SE204–SE206 (**c**), and SE207 (**d**). Data are normalized to the native relaxin-2 response at human RXFP1 and are mean ± s.e.m. from technical triplicates.

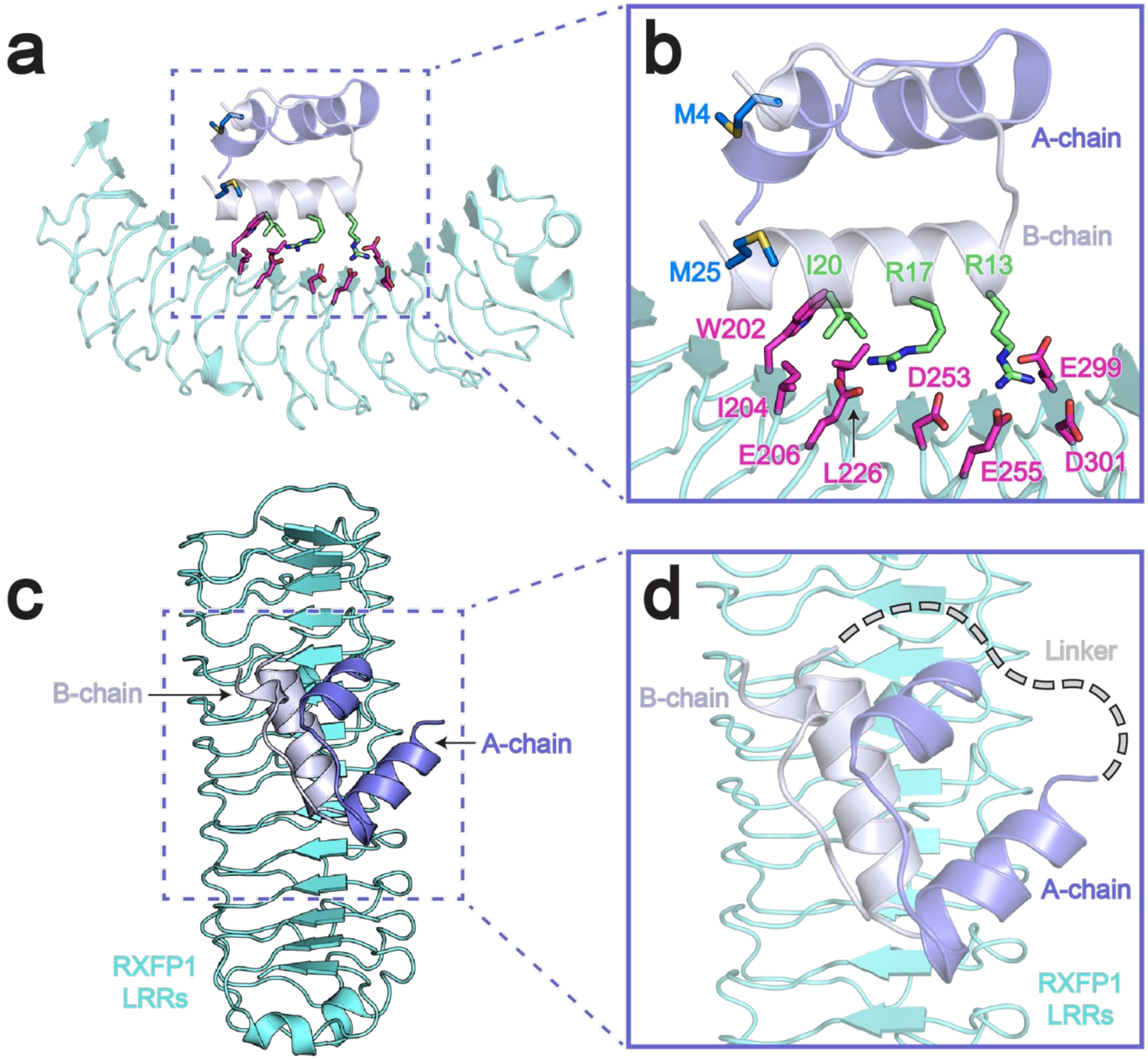

Figure S3: Sites of relaxin-2 engineering in Fc–relaxin-2 fusion constructs. a,**c**, Docking model of relaxin-2 bound to the leucine-rich repeats (LRRs) of RXFP1's ectodomain [1]. **b**, Details of the relaxin-2–LRR interface. In magenta are RXFP1 residues involved in the binding interface, in green are the "relaxin-binding cassette" residues of relaxin-2's B-chain, Arg13, Arg17, and Ile20, and in blue are the Met residues mutated to Lys in SE301. The Trp28 residue was not included in the docking model. The model shows that the Met residues on the relaxin-2 B-chain are not positioned near the binding interface. **d**, Based on the model, the position of the single-chain relaxin-2 "mini-C" linker likely does not interfere with the binding of relaxin-2 to RXFP1.

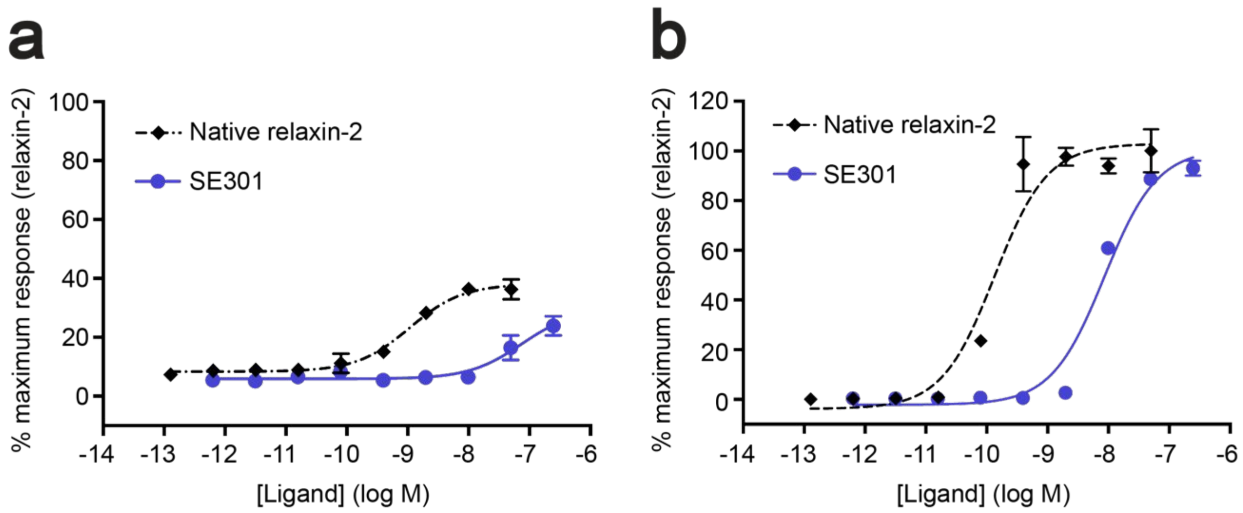

Figure S4: SE301 signaling activity at human RXFP2 and mouse RXFP1. a, CRE-SEAP G_s signaling assay data for SE301 compared to native relaxin-2 at human RXFP2. Data are normalized to the native relaxin-2 response at human RXFP1 and are mean ± s.e.m. from technical triplicates. **b**, CRE-SEAP G_s signaling assay data for SE301 compared to native human relaxin-2 at mouse RXFP1. Data are normalized to the native human relaxin-2 response at mouse RXFP1 and are mean ± s.e.m. from technical triplicates.

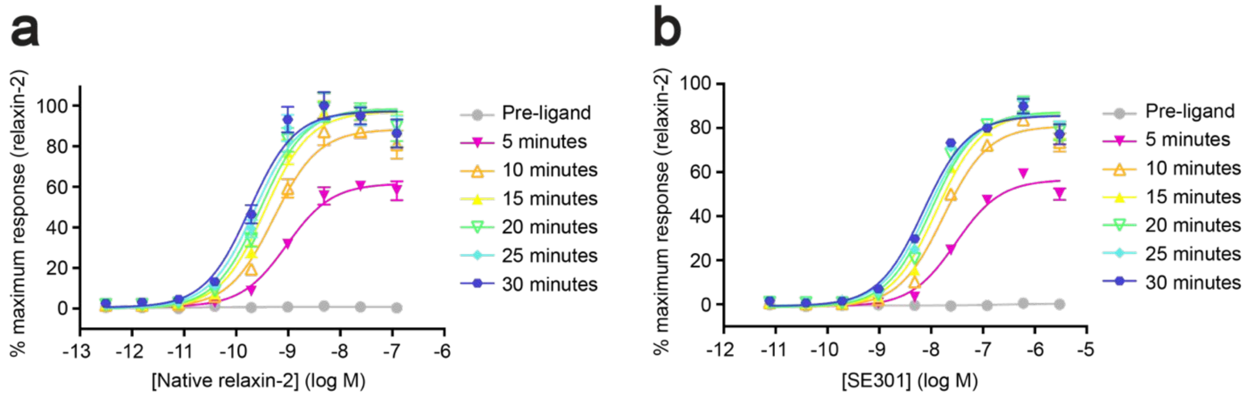

Figure S5: SE301 versus native relaxin-2 activity in the G_s GloSensor cAMP assay. a-b, GloSensor G_s signaling assay data for native relaxin-2 (**a**) and SE301 (**b**) with measurements taken before ligand addition, and 5, 10, 15, 20, 25, and 30 minutes after ligand addition. Data are normalized to the native relaxin-2 response at human RXFP1 and are mean ± s.e.m. from technical triplicates.

Table S1: CRE-SEAP G_s signaling assay EC_{50} and E_{max} data for Figure 1c

†Mean ± s.e.m., n=3 technical replicates.

Ligand	pEC_{50}	E_{max} (%)
Native relaxin-2	10.0 ± 0.06	100 ± 2.0
SE301	8.2 ± 0.05	110 ± 2.5
Fc N297Q	ND	ND

Table S2: CRE-SEAP G_s signaling assay EC_{50} and E_{max} data for Figure S1c

†Mean ± s.e.m., n=3 technical replicates.

Ligand	pEC_{50}	E_{max} (%)
Native relaxin-2	10.0 ± 0.2	100 ± 6.9
SE001	9.1 ± 0.1	126 ± 3.3
SE004	8.8 ± 0.1	124 ± 2.9

Table S3: CRE-SEAP G_s signaling assay EC_{50} and E_{max} data for Figure S2

†Mean ± s.e.m., n=3 technical replicates.

Ligand	pEC_{50}	E_{max} (%)
Native relaxin-2	10.3 ± 0.1	100 ± 3.1
SE101	7.3 ± 0.1	86 ± 7.4
SE102	8.1 ± 0.1	106 ± 3.9
SE103	7.7 ± 0.1	93 ± 4.5
Native relaxin-2	10.5 ± 0.1	100 ± 3.5
SE201	8.4 ± 0.1	102 ± 3.1
SE202	8.4 ± 0.1	110 ± 3.3
SE203	8.2 ± 0.1	122 ± 3.0
SE204	8.8 ± 0.1	116 ± 3.3
SE205	9.3 ± 0.1	132 ± 3.1
SE206	8.5 ± 0.1	138 ± 4.9
SE207	8.4 ± 0.1	122 ± 3.3

Table S4: CRE-SEAP G_s signaling assay EC_{50} and E_{max} data for Figure S4

†Mean ± s.e.m., n=3 technical replicates.

Ligand	pEC_{50}	E_{max} (%)
Native relaxin-2, human RXFP2	9.0 ± 0.1	38 ± 1.7
SE301, human RXFP2	7.1 ± 0.3	30 ± 5.7
Native relaxin-2, mouse RXFP1	9.9 ± 0.1	100 ± 4.2
SE301, mouse RXFP1	8.1 ± 0.1	98 ± 3.7

Table S5: GloSensor G_s signaling assay EC_{50} and E_{max} data for Figure S5

†Mean ± s.e.m., n=3 technical replicates.

Ligand	pEC_{50}	E_{max} (%)
Native relaxin-2, Pre-ligand	ND	1 ± 0.2
Native relaxin-2, 5 minutes	9.0 ± 0.1	63 ± 1.7
Native relaxin-2, 10 minutes	9.3 ± 0.1	91 ± 2.7
Native relaxin-2, 15 minutes	9.4 ± 0.1	100 ± 2.9
Native relaxin-2, 20 minutes	9.5 ± 0.1	101 ± 2.9
Native relaxin-2, 25 minutes	9.6 ± 0.1	100 ± 3.2
Native relaxin-2, 30 minutes	9.7 ± 0.1	100 ± 3.1
SE301, Pre-ligand	ND	0 ± 0.5
SE301, 5 minutes	7.5 ± 0.1	58 ± 1.6
SE301, 10 minutes	7.8 ± 0.1	83 ± 2.0
SE301, 15 minutes	7.9 ± 0.1	89 ± 2.0
SE301, 20 minutes	8.0 ± 0.1	90 ± 2.2
SE301, 25 minutes	8.1 ± 0.1	89 ± 2.2
SE301, 30 minutes	8.1 ± 0.1	88 ± 2.0

Table S6: CRE-SEAP G_s signaling assay EC_{50} and E_{max} data for Figure 3b

†Mean ± s.e.m., n=3 technical replicates.

Ligand	pEC_{50}	E_{max} (%)
Native relaxin-2	9.9 ± 0.05	100 ± 1.7
SE301 Day 0	8.1 ± 0.05	102 ± 2.4
SE301 Day 7	8.2 ± 0.03	104 ± 1.3
SE301 Day 14	8.1 ± 0.1	98 ± 2.4
SE301 Day 21	8.1 ± 0.04	98 ± 2.0
SE301 Day 28	8.3 ± 0.04	108 ± 2.1

Supplementary references

1. Erlandson, S. C. *et al.* The relaxin receptor RXFP1 signals through a mechanism of autoinhibition. *Nat Chem Biol* **19**, 1013–1021 (2023).

Table S7: Construct sequences.

Name	Sequence
**SE001**	MKTIIALSYIFCLVFAHHHHHHDSWMEEVIKLCGRELVRAQIAICGMSTWSDAASSHSHSSARQLYSALANKCCHVGCTKRSLARFC
**SE004**	MKTIIALSYIFCLVFAEDQVDPRLIDGKGSHHHHHHDSWMEEVIKLCGRELVRAQIAICGMSTWSDAASSHSHSSARQLYSALANKCCHVGCTKRSLARFC
**SE101**	MYRMQLLSCIALSLALVTNSDSWMEEVIKLCGRELVRAQIAICGMSTWSDAASSHSHSSARQLYSALANKCCHVGCTKRSLARFCGSGGSGDKTHTCPPCPAPELLGGPSVFLFPPKPKDTLMISRTPEVTCVVVDVSHEDPEVKFNWYVDGVEVHNAKTKPREEQYNSTYRVVSVLTVLHQDWLNGKEYKCKVSNKALPAPIEKTISKAKGQPREPQVYTLPPSREEMTKNQVSLTCLVKGFYPSDIAVEWESNGQPENNYKTTPPVLDSDGSFFLYSKLTVDKSRWQQGNVFSCSVMHEALHNHYTQKSLSLSPGK
**SE102**	MKTIIALSYIFCLVFADKTHTCPPCPAPELLGGPSVFLFPPKPKDTLMISRTPEVTCVVVDVSHEDPEVKFNWYVDGVEVHNAKTKPREEQYNSTYRVVSVLTVLHQDWLNGKEYKCKVSNKALPAPIEKTISKAKGQPREPQVYTLPPSREEMTKNQVSLTCLVKGFYPSDIAVEWESNGQPENNYKTTPPVLDSDGSFFLYSKLTVDKSRWQQGNVFSCSVMHEALHNHYTQKSLSLSPGKGGSDSWMEEVIKLCGRELVRAQIAICGMSTWSDAASSHSHSSARQLYSALANKCCHVGCTKRSLARFC
**SE103**	MKTIIALSYIFCLVFADKTHTCPPCPAPELLGGPSVFLFPPKPKDTLMISRTPEVTCVVVDVSHEDPEVKFNWYVDGVEVHNAKTKPREEQYNSTYRVVSVLTVLHQDWLNGKEYKCKVSNKALPAPIEKTISKAKGQPREPQVYTLPPSREEMTKNQVSLTCLVKGFYPSDIAVEWESNGQPENNYKTTPPVLDSDGSFFLYSKLTVDKSRWQQGNVFSCSVMHEALHNHYTQKSLSLSPGKGGSGGSGGSGGSDSWMEEVIKLCGRELVRAQIAICGMSTWSDAASSHSHSSARQLYSALANKCCHVGCTKRSLARFC
**SE201**	MKTIIALSYIFCLVFADKTHTCPPCPAPELLGGPSVFLFPPKPKDTLMISRTPEVTCVVVDVSHEDPEVKFNWYVDGVEVHNAKTKPREEQY**Q**STYRVVSVLTVLHQDWLNGKEYKCKVSNKALPAPIEKTISKAKGQPREPQVYTLPPSREEMTKNQVSLTCLVKGFYPSDIAVEWESNGQPENNYKTTPPVLDSDGSFFLYSKLTVDKSRWQQGNVFSCSVMHEALHNHYTQKSLSLSPGKGGSDSWMEEVIKLCGRELVRAQIAICGMSTWSDAASSHSHSSARQLYSALANKCCHVGCTKRSLARFC
**SE202**	MKTIIALSYIFCLVFADKTHTCPPCPAPELLGGPSVFLFPPKPKDTLMISRTPEVTCVVVDVSHEDPEVKFNWYVDGVEVHNAKTKPREEQY**Q**STYRVVSVLTVLHQDWLNGKEYKCKVSNKALPAPIEKTISKAKGQPREPQVYTLPPSREEMTKNQVSLTCLVKGFYPSDIAVEWESNGQPENNYKTTPPVLDSDGSFFLYSKLTVDKSRWQQGNVFSCSVMHEALHNHYTQKSLSLSPGKAAADSWMEEVIKLCGRELVRAQIAICGMSTWSDAASSHSHSSARQLYSALANKCCHVGCTKRSLARFC
**SE203**	MKTIIALSYIFCLVFADKTHTCPPCPAPELLGGPSVFLFPPKPKDTLMISRTPEVTCVVVDVSHEDPEVKFNWYVDGVEVHNAKTKPREEQY**Q**STYRVVSVLTVLHQDWLNGKEYKCKVSNKALPAPIEKTISKAKGQPREP

	QVYTLPPSREEMTKNQVSLTCLVKGFYPSDIAVEWESNGQPENNYKTTPPVLDSDGSFFLYSKLTVDKSRW<br>QQGNVFSCSVMHEALHNHYTQKSLSLSPGKPPPDSWMEEVIKLCGRELVRAQIAICGMSTWSDAASSHSH<br>SSARQLYSALANKCCHVGCTKRSLARFC
**SE204**	MKTIIALSYIFCLVFADKTHTCPPCPAPELLGGPSVFLFPPKPKDTLMISRTPEVTCVVVDVSHEDPEVKFNW<br>YVDGVEVHNAKTKPREEQY**Q**STYRVVSVLTVLHQDWLNGKEYKCKVSNKALPAPIEKTISKAKGQPREP<br>QVYTLPPSREEMTKNQVSLTCLVKGFYPSDIAVEWESNGQPENNYKTTPPVLDSDGSFFLYSKLTVDKSRW<br>QQGNVFSCSVMHEALHNHYTQKSLSLSPGKGGSDSW**K**EEVIKLCGRELVRAQIAICGMSTWSDAASSHSH<br>SSARQLYSALANKCCHVGCTKRSLARFC
**SE205**	MKTIIALSYIFCLVFADKTHTCPPCPAPELLGGPSVFLFPPKPKDTLMISRTPEVTCVVVDVSHEDPEVKFNW<br>YVDGVEVHNAKTKPREEQY**Q**STYRVVSVLTVLHQDWLNGKEYKCKVSNKALPAPIEKTISKAKGQPREP<br>QVYTLPPSREEMTKNQVSLTCLVKGFYPSDIAVEWESNGQPENNYKTTPPVLDSDGSFFLYSKLTVDKSRW<br>QQGNVFSCSVMHEALHNHYTQKSLSLSPGKGGSDSWMEEVIKLCGRELVRAQIAICG**K**STWSDAASSHSH<br>SSARQLYSALANKCCHVGCTKRSLARFC
**SE206**	MKTIIALSYIFCLVFADKTHTCPPCPAPELLGGPSVFLFPPKPKDTLMISRTPEVTCVVVDVSHEDPEVKFNW<br>YVDGVEVHNAKTKPREEQY**Q**STYRVVSVLTVLHQDWLNGKEYKCKVSNKALPAPIEKTISKAKGQPREP<br>QVYTLPPSREEMTKNQVSLTCLVKGFYPSDIAVEWESNGQPENNYKTTPPVLDSDGSFFLYSKLTVDKSRW<br>QQGNVFSCSVMHEALHNHYTQKSLSLSPGKGGSDSWMEEVIKLCGRELVRAQIAICGMST**A**SDAASSHSH<br>SSARQLYSALANKCCHVGCTKRSLARFC
**SE207**	MKTIIALSYIFCLVFADKTHTCPPCPAPELLGGPSVFLFPPKPKDTLMISRTPEVTCVVVDVSHEDPEVKFNW<br>YVDGVEVHNAKTKPREEQY**Q**STYRVVSVLTVLHQDWLNGKEYKCKVSNKALPAPIEKTISKAKGQPREP<br>QVYTLPPSREEMTKNQVSLTCLVKGFYPSDIAVEWESNGQPENNYKTTPPVLDSDGSFFLYSKLTVDKSRW<br>QQGNVFSCSVMHEALHNHYTQKSLSLSPGKGGSDSWMEEVIKLCGRELVRAQIAICGMSTWSDAA**GANA**<br>**NAG**ARQLYSALANKCCHVGCTKRSLARFC
**SE301**	MKTIIALSYIFCLVFADKTHTCPPCPAPELLGGPSVFLFPPKPKDTLMISRTPEVTCVVVDVSHEDPEVKFNW<br>YVDGVEVHNAKTKPREEQY**Q**STYRVVSVLTVLHQDWLNGKEYKCKVSNKALPAPIEKTISKAKGQPREP<br>QVYTLPPSREEMTKNQVSLTCLVKGFYPSDIAVEWESNGQPENNYKTTPPVLDSDGSFFLYSKLTVDKSRW<br>QQGNVFSCSVMHEALHNHYTQKSLSLSPGKGGSDSW**K**EEVIKLCGRELVRAQIAICG**K**ST**A**SDAA**GANAN**<br>**AG**ARQLYSALANKCCHVGCTKRSLARFC
**Fc N297Q**	MKTIIALSYIFCLVFADKTHTCPPCPAPELLGGPSVFLFPPKPKDTLMISRTPEVTCVVVDVSHEDPEVKFNW<br>YVDGVEVHNAKTKPREEQY**Q**STYRVVSVLTVLHQDWLNGKEYKCKVSNKALPAPIEKTISKAKGQPREP<br>QVYTLPPSREEMTKNQVSLTCLVKGFYPSDIAVEWESNGQPENNYKTTPPVLDSDGSFFLYSKLTVDKSRW<br>QQGNVFSCSVMHEALHNHYTQKSLSLSPGK

Legend: Signal sequence, His tag, Protein C tag, Relaxin-2 A-chain, Relaxin-2 B-chain, Fc, **Mutation**
